# Unravelling the intricate cooperativity of subunit gating in P2X2 ion channels

**DOI:** 10.1038/s41598-020-78672-w

**Published:** 2020-12-10

**Authors:** Christian Sattler, Thomas Eick, Sabine Hummert, Eckhard Schulz, Ralf Schmauder, Andrea Schweinitz, Christopher Unzeitig, Frank Schwede, Klaus Benndorf

**Affiliations:** 1 Institut für Physiologie II, Universitätsklinikum Jena, Friedrich-Schiller-Universität Jena, Kollegiengasse 9, 07743 Jena, Germany; 2 Fakultät Elektrotechnik, Fachhochschule Schmalkalden, Blechhammer, 98574 Schmalkalden, Germany; 3 BIOLOG Life Science Institute GmbH & Co. KG, Flughafendamm 9A, 28199 Bremen, Germany

**Keywords:** Ion channels, Computational biophysics, Molecular biophysics, Biophysics, Chemical biology, Physiology, Neuroscience, Ion channels in the nervous system

## Abstract

Ionotropic purinergic (P2X) receptors are trimeric channels that are activated by the binding of ATP. They are involved in multiple physiological functions, including synaptic transmission, pain and inflammation. The mechanism of activation is still elusive. Here we kinetically unraveled and quantified subunit activation in P2X2 receptors by an extensive global fit approach with four complex and intimately coupled kinetic schemes to currents obtained from wild type and mutated receptors using ATP and its fluorescent derivative 2-[DY-547P1]-AET-ATP (fATP). We show that the steep concentration-activation relationship in wild type channels is caused by a subunit flip reaction with strong positive cooperativity, overbalancing a pronounced negative cooperativity for the three ATP binding steps, that the net probability fluxes in the model generate a marked hysteresis in the activation-deactivation cycle, and that the predicted fATP binding matches the binding measured by fluorescence. Our results shed light into the intricate activation process of P2X channels.

## Introduction

Ionotropic purinergic receptors (P2X receptors) are expressed in many tissues and they are involved in diverse physiological and pathophysiological processes like pain, inflammation, taste or synaptic transmission^1,2^. The channels are activated by the binding of ATP at their extracellular side and they generate a non-specific cation conductance^3^. Seven mammalian subunit isoforms have been identified^4,5^. The crystal structure of the zebrafish P2X4 (zfP2X4) channel has proven a trimeric architecture and revealed that the shape of a single subunit resembles that of a dolphin^6,7^, which was later confirmed for P2X3 receptors^8^. The transmembrane domains TM1 and TM2 form the tail and the ensemble of the three TM2 helices in a channel builds the pore^9^. The large ectodomain is organized in a β-sheet structure with lateral fenestration sites. From this body four structurally flexible domains ramify, including head, dorsal fin, left and right flipper. ATP is bound to binding sites between two neighbored subunits, distant by as much as 40 Å from the extracellular boundary of the transmembrane domain, and cradled by the dolphin head, upper body, lower body, left flipper and dorsal fin.

It is presently not fully clear how the signal of ligand binding propagates and opens the channel pore. Two studies suggested that two activated subunits suffice for channel activation^10–12^ whereas other studies provide evidence that all three subunits are involved^10,13–15^. For transmission the signal of ATP binding to the pore opening, a central role of the β-14 sheet has been suggested, connecting within a subunit the ATP binding site on the upper end with the pore forming TM2 helix on the lower end, thereby interacting with the β-1 sheet of the adjacent subunit^16^. Regarding the subunit interaction, it has been proposed that each ATP-binding signal propagates first along the same subunit, then spreads equally to all three subunits towards the pore^11^ and results in a symmetrical closed-open transition^17^. Another study distinguishes five key steps in activation^18^. However, many questions remain open, including the type of subunit cooperativity and propagation of ligand binding to the opening of the gate.

Major progress in understanding the function of P2X receptors can be expected from applying global fit analyses of extended data, as demonstrated previously for other ion channels. Using current data at equilibrium this has been demonstrated for concentration-activation relationships of concatenated CNGA2 channels, containing a different number of disabled binding sites^19^, for *IV*-relationships of viral Kv channels at different concentrations of permeating K^+^ ions^20^, or gating charge movement in Shaker channels^21^. We previously also globally fitted data distant from equilibrium by kinetic schemes, for activation time courses of tetrameric CNGA2 channels induced by cGMP jumps to different concentrations^22^ and voltage-induced activation time courses of tetrameric HCN2 channels^23^. Moreover, we extended these analyses by combining electrophysiological time-dependent data with the corresponding orthogonal data of ligand binding which enabled us to specify kinetic schemes in considerable detail and to identify complex types of cooperativity among the subunits in CNGA2^24,25^ and HCN2 channels^26^.

Herein we analyzed ATP-induced activation of P2X2 receptors in great detail by a novel global fit approach with extended and orthogonal data. We included both equilibrium and non-equilibrium data at multiple ligand concentrations, data from the facilitating mutant H319K in the transmission pathway, and data obtained with an ATP-derivative with significantly lower apparent affinity 2-[DY-547P1]-AET-ATP (fATP)^27^. We globally fitted these heterogeneous data with four complex kinetic schemes, each including three binding reactions, three flip and three open reactions and, importantly, the four models were intimately coupled by sharing the majority of parameters. These results led us identify and quantify a simple, and also surprising, cooperativity pattern for the wild type channels and recognize that the steep concentration–activation relationship in P2X2 channels is the result of a subunit flip reaction with pronounced positive cooperativity that overbalances pronounced negative cooperativity for the three ATP binding steps. Moreover, it is shown how the mutation H319K promotes opening by significantly facilitating the conformational flip in the channel liganded by only one ATP.

## Results

### Open probability in macroscopic currents

**Figure 1:**
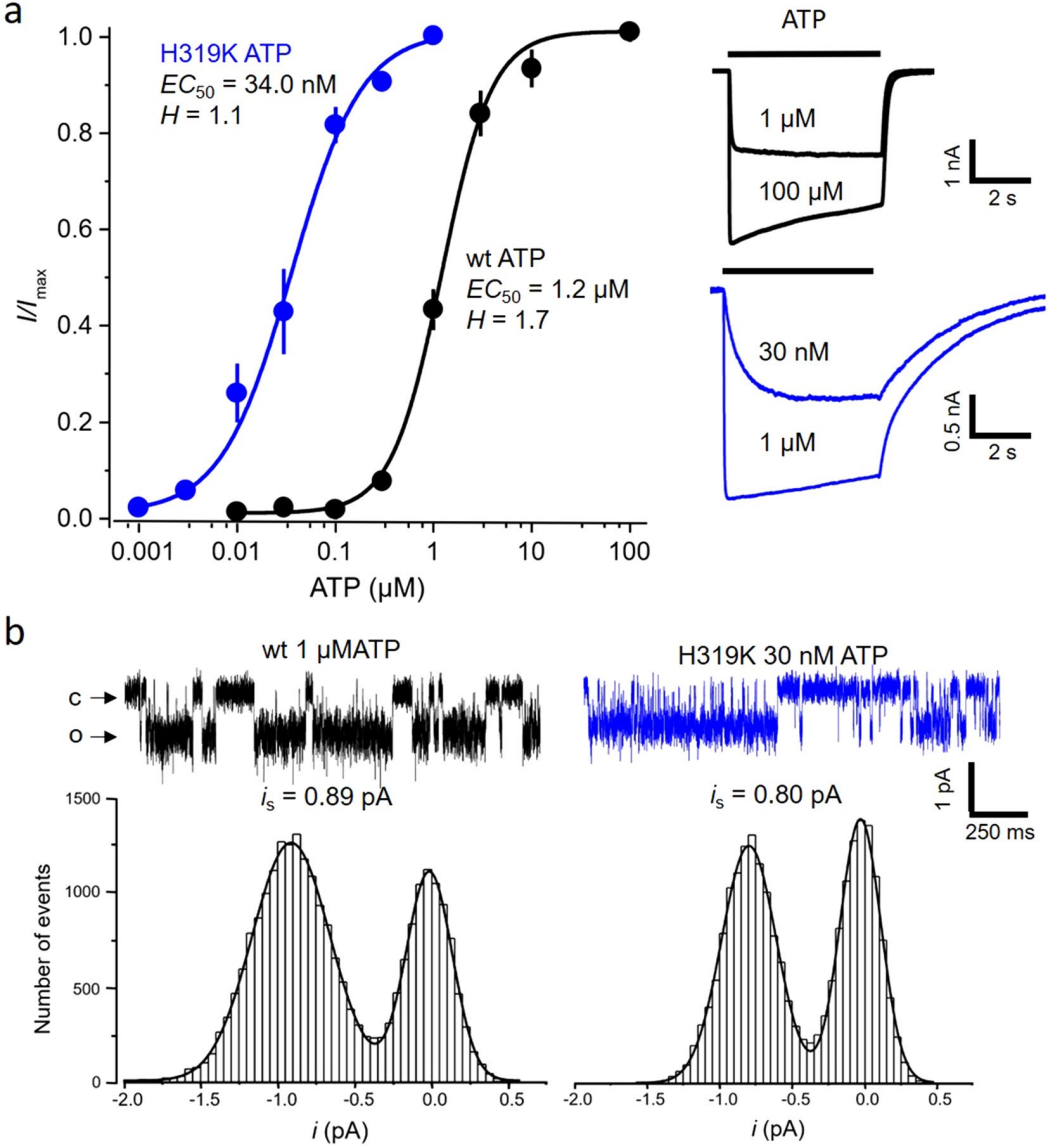
Currents in wild type and H319K channels. The membrane voltage was generally − 50 mV. (**a**) Concentration-activation relationships for wt (black) and H319K currents (blue) measured in whole cells. The data points were fitted with Hill curves according to Eq. (4) yielding the indicated parameters. The insets right show typical current time courses evoked by ATP pulses at the indicated concentrations. (**b**) Single-channel currents measured in the cell-attached configuration. Representative traces and amplitude histograms to determine the amplitude of the single-channel current *i*_s_ (see “Methods”).

All recordings subjected to the global fit analysis were performed in whole HEK293 cells at − 50 mV to stay in the physiological range of voltages and to obtain currents of reasonable amplitude. Solution jumps were administered by a fast application system, enabling the application of multiple concentrations to the same cell. The evoked currents were activated within hundreds of milliseconds to seconds and deactivated when jumping back to control solution (Fig. 1a). Slow desensitization in the presence of ATP, mostly pronounced at high ligand concentrations, was eliminated by including only the currents until the time of peak. The peak currents were related to those at saturating ATP to obtain the normalized concentration-activation-relationships (Fig. 1a; see “Methods”).

For the modeling below, the value of the open probability at saturating ATP, *P*_o,max_, was required to translate all ensemble currents to time courses of the open probability, *P*_o_, at the actual ATP concentration. *P*_o,max_ was determined from evaluating the noise variance *σ*^2^ near the peak current *I*_max_ at saturating ATP, which was corrected by the variance of the baseline, according to

1 $$P_{o,\max } = 1 - \sigma^{2} /iI_{\max } .$$

Herein, *i* is the amplitude of the single-channel current that was determined experimentally. The value was 0.80 pA ± 0.03 pA (n = 6) (Fig. 1b), resulting in a *P*_o,max_ value of 0.695 ± 0.009 (n = 6). With this value the concentration-activation relationship was translated to a concentration-*P*_o_ relationship to be used in the global fit approach.

### Model selection

**Figure 2:**
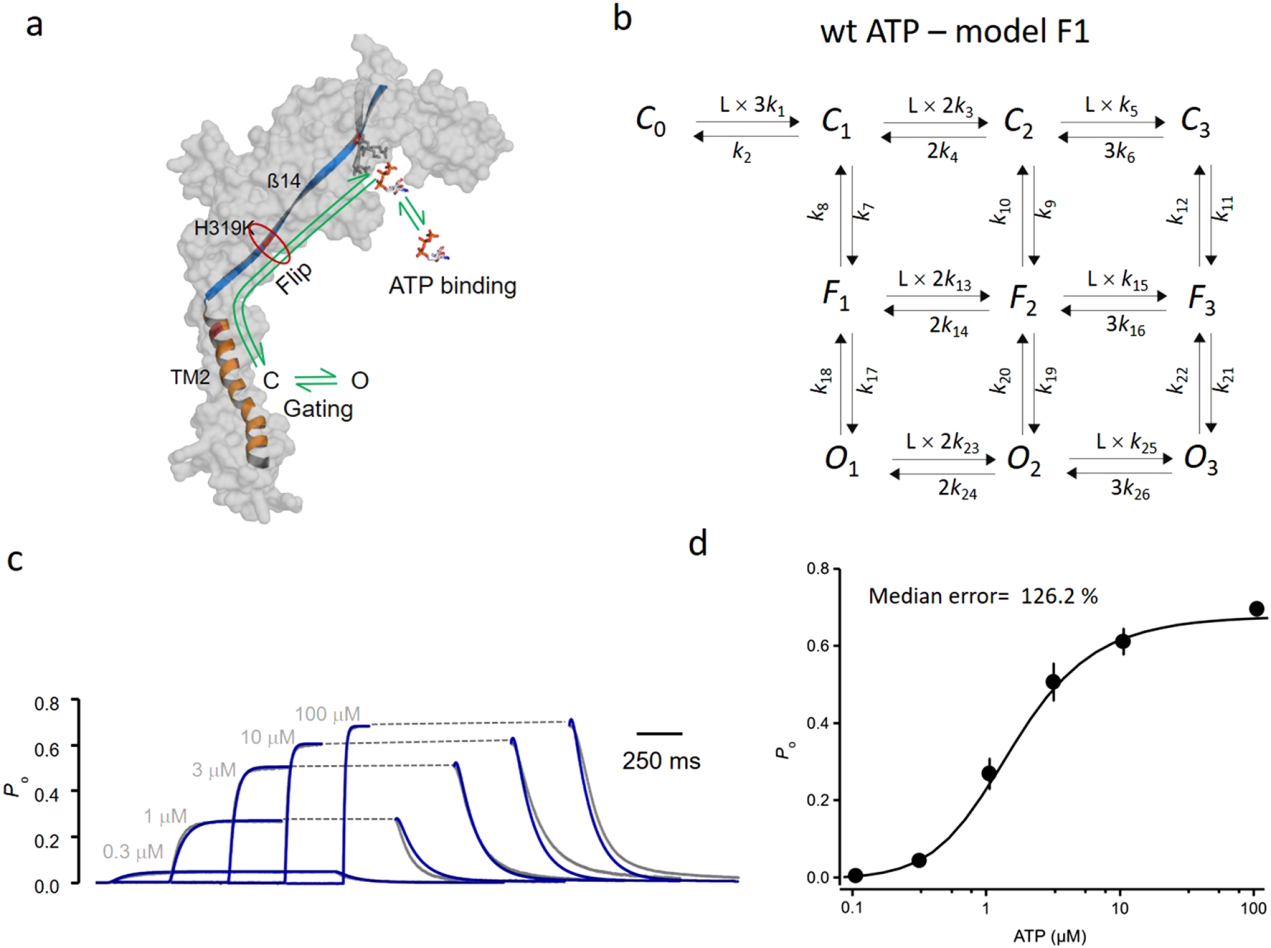
Global fit strategy. (**a**) Allocation of the processes governing the activation gating in P2X channels to the structure. As illustrated for one subunit, channel activation is composed of three steps including ATP binding, propagation of the binding signal by a conformational flip to the transmembrane gate, and gate opening. In the trimeric channel, interactions of the subunits have to be included. The subunit structure of P2X2 has been built as homology model to P2X3^8,27^. (**b**) Scheme of model F1 with three binding steps, (*C*_x−1_ − *C*_x_), respective flip reactions, *C*_x_ − *F*_x_, and flipped-open isomerizations, *F*_x_ − *O*_x_ (x = 1,2,3), i.e. ligand binding and unbinding proceeds in horizontal direction whereas the conformational flip (*C*_x_ − *F*_x_) and the closed-open isomerization (*F*_x_ − *O*_x_) in vertical direction. *k*_x_ defines a rate constant for a respective transition. The values of the rate constants are provided by Supplementary Table 1. (**c**) Activation and deactivation time courses (gray) of wt channels evoked by ATP jumps at the indicated concentrations. The time courses were globally fitted (blue curves) together with concentration-activation relationship (**d**) with model F1 shown in b.

We considered only models with three functionally relevant binding steps because (1.) the structure reveals three ATP binding sites, located in the large extracellular domain distant from the gate in the TMD (Fig. 2a), and (2.) P2X2 concatamers with selectively disabled binding sites show a proportional increase of the current density with the number of functional binding sites^10^. Accordingly, our initial model comprised three binding steps followed by an open step at each degree of ligand binding (model 1; Supplementary Fig. 1a ). Furthermore, for the unliganded channel we assumed that no opening proceeds because there is no experimental evidence for relevant channel activity in the absence of ATP. Since it is a priori not clear to what extent the subunits interact upon activation, fixed stoichiometries of the rate constants, statistically weighted according to the number of associating or dissociating ligands, were avoided. Model 1 with the direct closed-open transitions clearly failed to describe the data: It generated about five times slower activation time courses at intermediate concentrations compared to the measurements. Moreover, the fit produced a pronounced upward deflection in the early deactivation time courses that was not present in the data.

To overcome these mismatches, we extended our model by introducing flipped states in the closed-open isomerisations (Model F1; Fig. 2b). This extension improved the fit significantly in both the activation time courses and early deactivation (Fig. 2c,d). Because ligand binding appears distant from the channel gate (Fig. 2a), it seemed to us a plausible assumption to attribute the binding steps of the model to processes at the binding sites, the flipped steps to conformational changes propagating from the binding sites to the gate, and the opening step to a closed-open isomerization in the transmembrane helix TM2. However, despite the improvement of the fit, many errors of the parameters exceeded 100% (Supplementary Table 1), precluding any interpretation of the resulting rate constants. Hence, our approach to globally fit time courses and the concentration-*P*_o_ relationship at equilibrium for wild type channels and ATP is insufficient.

### Extending the global fit strategy

**Figure 3:**
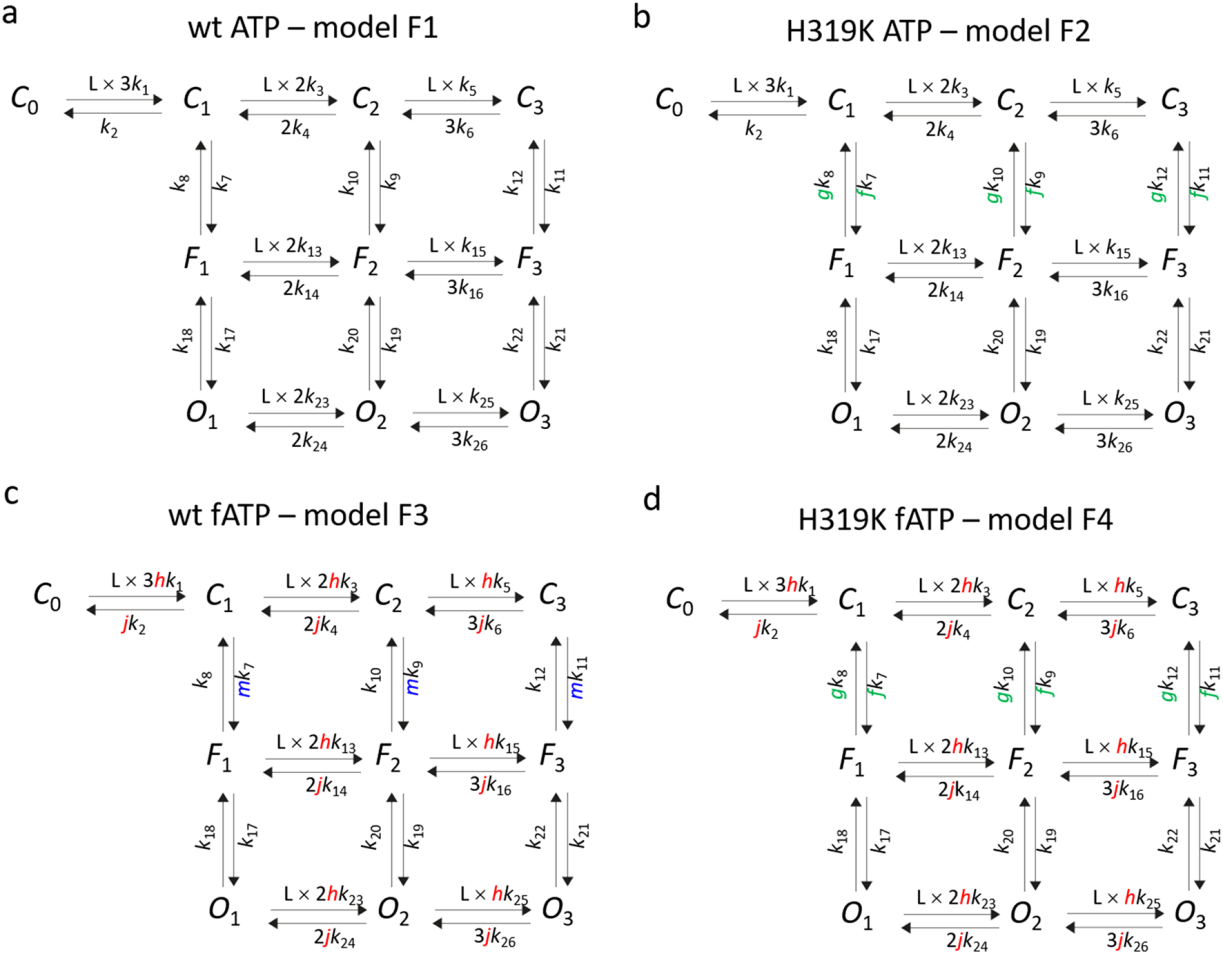
Models used for the extended global fits. In addition to model F1, the models F2, F3 and F4 were used to fit the data of H319K ATP, wt fATP and H319K fATP, respectively. The factors *f*, *g*, *h*, and *j* are coupling factors generating functional differences in the models. *f* and *g* were used to accelerate and decelerate the *C*_x_ → *F*_x_ and *F*_x_ → *C*_x_ steps whereas *hC*_x_ → *F*_x_ step of model F3 to enable fitting of the concentration-*P*_o_ relationship. For further explanation see text.

In order to increase the constraint conditions and enhance model predictability, we gathered more functional information. We therefore included in our analysis P2X2 channels carrying the mutation H319K, a mutation that has been reported to enhance the apparent affinity of P2X2 channels by a factor of 40^28^. Naturally, the histidine, which is unique in P2X2 receptors and located in the upper part of the β-14 linker, is responsible for the positive modulation of protons on ATP induced currents via an allosteric binding site. Since the distance of H319 to all three binding sites on the one end and the gate on the other is similarly long and the β-14 linker is well in that part of the channel structure propagating the binding information to the pore, we assume that in our model this mutation affects the flip step (c.f. Figure 2a). Accordingly, a similar mechanism was attributed by the single amino acid P121 in the ε-subunit of nicotinic acetylcholine receptors^29^. We further assume that in model F1 all three flipping rates *C*_x_ → *F*_x_ are accelerated by the same factor *f* whereas the reverse reactions *F*_x_ → *C*_x_ are decelerated by the factor *g*, yielding model F2. All other rate constants in model F1 and F2 were the same (Fig. 3b; Supplementary Fig. 2). *P*_o,max_ in H319K channels was determined analogue as described above for wt channels. The single-channel current amplitude was determined to be 0.78 pA ± 0.05 pA (n = 5) (Fig. 1b) which was not statistically different (p = 0.66; *t*-test) from wt channels, resulting in a *P*_o,max_ value of 0.735 ± 0.054 (n = 7). For the modeling we therefore pooled the data and used the same maximum *P*_o,max_ = 0.719 ± 0.031 (n = 13). Then the intimately coupled models F1 and F2 were globally fitted to the two respective data sets (^2^global fit). The result with ^2^global fit was a new set of rate constants and, notably, that the relative error of the parameters was significantly smaller than fitting only model F1 to the wt ATP data with ^1^global fit (Supplementary Table 2).

**Figure 4:**
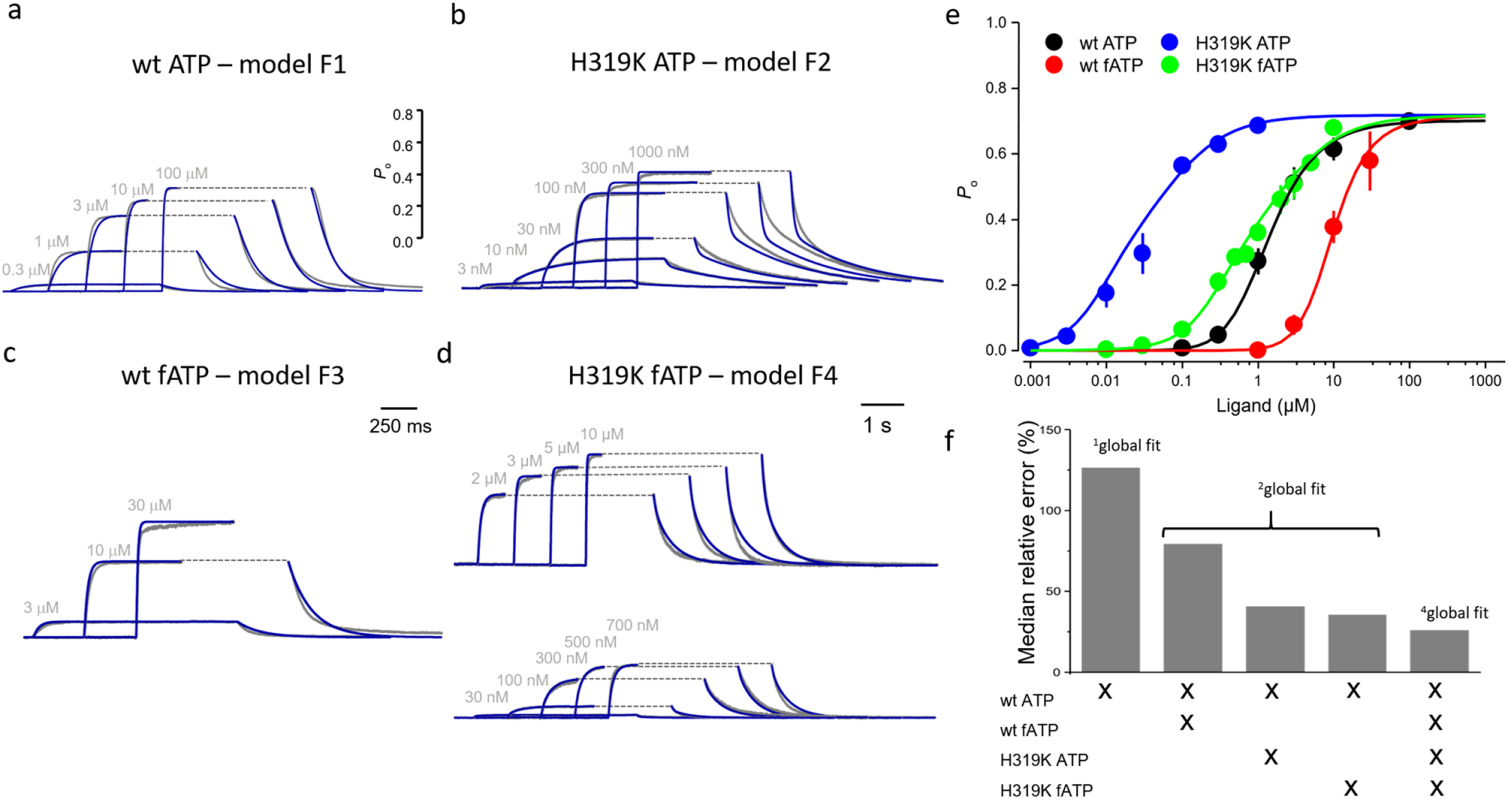
^4^Global fit. (**a**–**d**) Activation and deactivation time courses of the data (gray) wt ATP, H319K ATP, wt fATP, and H319K fATP. The respective ligand concentrations are indicated. (**e**) Equilibrium concentration-*P*_o_ relationships corresponding to the concentrations in (**a**–**d**). The data in (**a**–**e**) were globally fitted (**a**–**d**) blue curves; (**e**) colored curves) with the models F1, F2, F3 and F4 (Fig. 3) to the data by the ^4^global fit. **f** Median of the relative error of the parameters obtained by the indicated global fits. The ^4^global fit produced the lowest median of the relative error. For further explanation see text and “Methods”.

To further increase the experimental information, we modulated the binding process by using a second ligand with lower potency to activate the channels. We used the fluorescent 2-[DY-547P1]-AET-ATP (fATP)^27^ whose apparent affinity is 9 and 28 times reduced for wt and H319 channels, respectively. (The fact that fATP can report binding via the fluorescence intensity will allow us below to experimentally verify the calculated receptor occupancy.) For wt ATP and wt fATP we performed another ^2^global fit with model F1 and F3 by introducing the factor *h* and *j* in all binding and unbinding reactions (Fig. 3c; Supplementary Fig. 3), respectively, yielding a new set of rate constants (Supplementary Table 3). Again, all other rate constants were unchanged. The median relative error was also clearly improved compared to that obtained with ^1^global fit of wt ATP data only, but not as good as that obtained with the ^2^global fit for wt ATP and H319K ATP (Fig. 4f). Again, all other rate constants were unchanged. Further improvement of the median relative error was obtained when fitting the wt ATP data set in parallel to the H319K fATP data set by another ^2^global fit, using model F1 and F4 (Supplementary Fig. 4, Supplementary Table 4, Figs. 3d, 4d). Here the factors *f*, *h*, *g* and *j* were used together accordingly.

To further increase the determinateness of the parameters, it was finally intriguing to increase the constraints by performing a ^4^global fit of all four data sets, i.e. wt ATP, H319K ATP, wt fATP, and H319K fATP (Supplementary Fig. 5) with all four models (Fig. 3). Indeed, the median relative error became smaller again (Fig. 4f). However, the strongly increased constraints in the ^4^global fit produced a directly noticeable mismatch in the equilibrium concentration-*P*_o_ relationship of the wt fATP data, predicting less opening than found experimentally (red arrow in Supplementary Fig. 5). We therefore repeated the ^4^global fits with an additional factor to enhance the *P*_o_ at higher ligand concentrations. With a single factor, the only possibility turned out to accelerate *k*_7_, *k*_9_ *k*_11_, i.e. when accelerating the *C*_x_ → *F*_x_ rates. To this end we introduced in model F3 an additional factor *m* for these parameters and performed the fit de novo, yielding a reasonable description of the data (Fig. 4a–e). The errors of all rate constants were now in an acceptable range below 50% with the exception of *k*_21_ which was with 54% only slightly above (Supplementary Table 6). Accordingly, also the median relative error of the ^4^global fit was lowest (Fig. 4f). We like to emphasize that it is not the increased number of data points which has caused this gain of accuracy because, as described in “Methods”, the weight of the data points was artificially kept constant in all fits by appropriate factors.

The ^4^global fit with the coupling factors *f*, *g*, *h*, *j* and *m* and the resulting rate constants *k* for each transition gives us the opportunity for further interpretation of the model. It should be noted that our analysis provided us three specific factors for isomerisation steps in the model: *f* (11.20) accelerates the flip step from the closed to the open states in H319K whereas *g* (5.14 × 10^−4^) practically abolishes the respective backward reaction. *m* (15.59) evokes an accelerated flip in wt channels with fATP, a finding that is to some extent surprising. In contrast, *h* (0.17) and *j* (5.57) are modifiers of the binding and unbinding steps, respectively.

**Figure 5:**
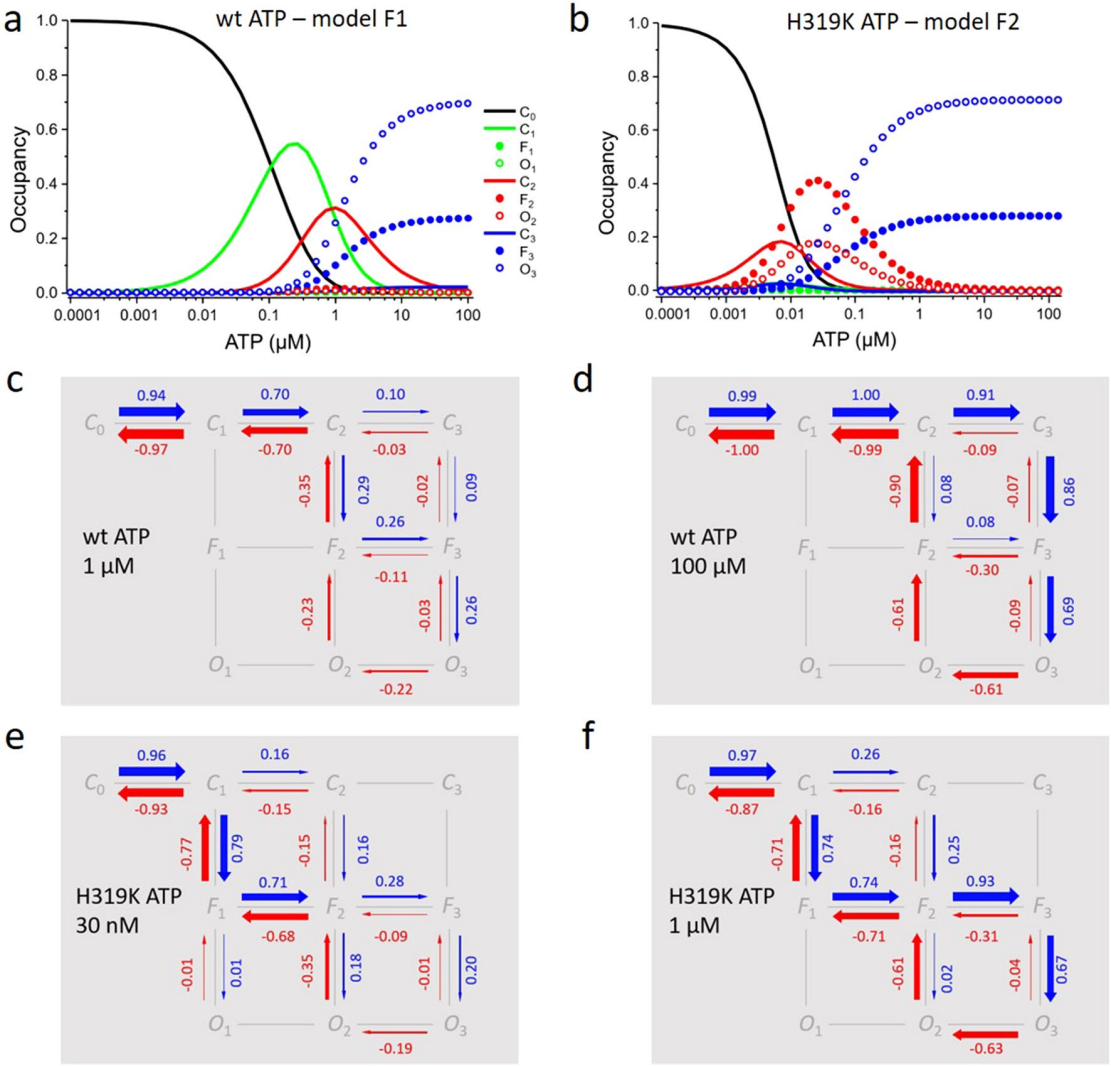
State occupancies at equilibrium and probability fluxes. (**a**) Occupancy of the states for wt channels as function of the ATP concentration according to model F1. (**b**) Occupancy of the states for H319K channels as function of the ATP concentration according to model F2. The partially liganded states *F*_2_ and *O*_2_ are significantly populated and contribute to the higher apparent affinity of the mutant. (**c**). Net probability fluxes in model F1 for wt channels when jumping the ATP concentration from zero to 1 μM (blue arrows) and back to zero (red arrows). The thickness of the arrows symbolizes the amount of net probability flux which is also specified by the adjacent numbers. Net probability fluxes below 0.01 were omitted. (**d**) As c but with 100 μM ATP. € Net probability fluxes in model F2 for H319K channels when jumping the ATP concentration from zero to 30 nM and back to zero. Same symbolism as in (**c**). (**f**) As (**e**) but with 1 μM ATP.

Notably, the rate constants *k*_21_ (*F*_3_-*O*_3_ transition) and *k*_22_ (*O*_3_-*F*_3_ transition) in the ^4^global fit are in the range of 10^11^ s^−1^ which is much faster than the speed limit of our recording system, and they can therefore not be physically interpreted. In an attempt to estimate the influence of these rate constants of the *F*_3_–*O*_3_ step on our conclusions, we computed time courses with the equations used in the ^4^global fit, thereby systematically reducing *k*_21_ by orders of magnitude while preserving the ratio between *k*_21_ and *k*_22_. Using all other determined rate constants, we then evaluated when χ^2^ increased by more than 1%. This was obtained only when *k*_21_ was reduced by as much as 8 orders of magnitude to 2.38 × 10^3^ s^−1^, resulting in *k*_22_ = 9.38 × 10^2^ s^−1^. These values are well in a physically reasonable range and represent an estimate for a lower border of these transitions. Assuming a speed limit for closed-open transitions of ~ 10^6^ s^−1^^30^, all other determined rate constants are slower. It should also be noted, that the rate constants of ligand binding at low concentrations are sufficiently slow to be resolved in our recordings. One of the rate constants of unbinding, *k*_24_, was much too slow to contribute to a time-dependent change in our measurements. Consequently, this unbinding process did not contribute to the probability fluxes in our probability flux analysis (see below, Fig. 5). The equilibrium constants determined herein seem to be in a realistic, i.e. physically reasonable range.

### Occupation of states as function of the ATP concentration at equilibrium

Knowing all constants in model F1 enabled us next to consider the occupancy of all 10 state as function of the ATP concentration in wt channels at equilibrium (Fig. 5a). Thereby, the equilibrium constant of the ultrafast *F*_3_-*O*_3_ transition was treated as all other equilibrium constants. At increasing ATP, *C*_0_ becomes depopulated to progressively fill *C*_1_ and *C*_2_ and finally to establish an equilibrium between *F*_3_ and *O*_3_. Neither the fully liganded closed state *C*_3_ nor the partially liganded flipped and open states, *F*_1_, *F*_2_, *O*_1_ and *O*_2_, play a relevant role. Because some of them must be passed for channel opening, they must be instable. In H319K channels the situation differs notably (Fig. 5b). Here at increasing ATP *C*_2_ becomes irrelevant and the double-liganded states *F*_2_ and *O*_2_ contribute at intermediate ATP concentrations of which *O*_2_ contributes significantly to *P*_o_. At further increasing ATP, again *F*_3_ and *O*_3_ dominate, similar to wt channels. Hence, populating the double-liganded states *F*_2_ and *O*_2_ leads to the higher apparent affinity of the H319K mutant.

### Net probability fluxes and transition pathways

Further insights into the channel gating can be gained by considering the probability fluxes in model F1 and F2 and identifying thus the transition pathways of the probability following jumps of the ATP concentration. Using the constants obtained by the ^4^global fit (Supplementary Table 6), we simulated the time courses of the probability *P*_X_(*t*) that an individual state X is occupied during the activation and deactivation gating and computed the total net probability fluxes between state X and Y, *F*_XY_, (see “Methods”). This approach allows to identify the transition pathways^31–33^ in the respective model for a given ligand step.

Consider first the net probability fluxes for wt channels after applying 1 μM ATP, a concentration near the *EC*_50_ value (Fig. 5c). The predominant pathway to the most relevant open state *O*_3_ runs along *C*_1_-*C*_2_-*F*_2_-*F*_3_, of which *F*_2_ is less stable than the others (c.f. Figure 5a). The pathway along *C*_3_ instead *F*_2_ is also used to some extent. When applying the saturating concentration of 100 μM ATP (Fig. 5d), the transition pathway runs predominantly through the closed (non-flipped) states and opening is exclusively generated by *O*_3_ via *F*_3_. A subordinate activation pathway runs along *F*_2_ instead of *C*_3_. The transition pathway for deactivation differs from the activation pathway notably. Independent whether activation was partial (Fig. 5c) or complete (Fig. 5d), it runs with high preference through the double-liganded states *O*_2_-*F*_2_-*C*_2_ to *C*_1_ and *C*_0_, generating a pronounced hysteresis in the gating regarding the double- and triple-liganded states.

In H319K the net probability fluxes differ noticeably. At 30 nM ATP, again a concentration near the *EC*_50_ value, the binding of already the first ligand generates a significant flip *C*_1_-*F*_1_, and via *F*_2_ and *F*_3_ the relevant open states *O*_2_ and *O*_3_, respectively, are populated (Fig. 5e). In contrast to wt channels, the saturating concentration of 1 μM ATP also favors the pathway *C*_1_-*F*_1_-*F*_2_-*F*_3_ to generate predominant opening from the triple liganded flipped state *F*_3_ (Fig. 5f). Notably, also at the same ATP concentration of 1 μM the net probability fluxes in wt and H319K channels differ upon activation due to the mutation.

Similar to wt channels the predominant transition pathway for deactivation in H319K channels differs notably from that of activation, predominantly at the high ATP concentration of 1 μM, where it runs along *O*_3_-*O*_2_-*F*_2_ in contrast to activation which employs *F*_3_, generating again pronounced hysteresis for the closed-open isomerization. In contrast to wt channels, in H319K channels the flipping runs predominantly in both activation and deactivation with a high preference along the pathway *C*_1_-*F*_1_-*F*_2_ (Fig. 5e,f), i.e. without hysteresis (c.f. Figure 5c,d).

### Equilibrium free energies and cooperativity

**Figure 6:**
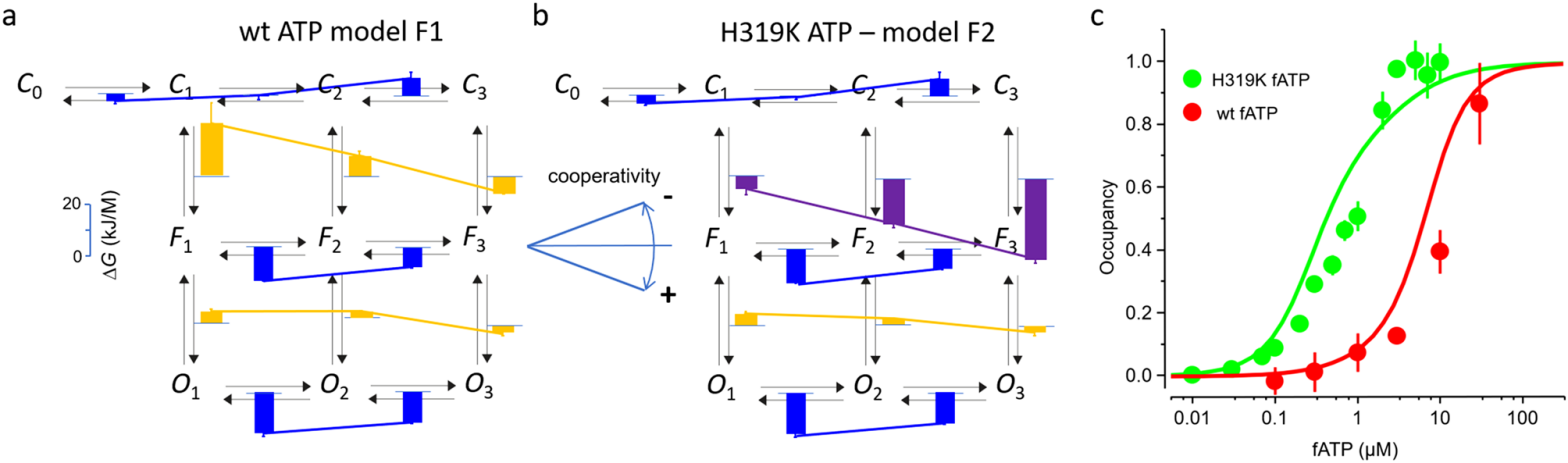
Gibbs free energies and predicted binding. (**a**) Gibbs free energy in model F1 and F2 for the association in the binding steps, Δ*G*_A_, at the ligand concentration *L* = 1 μM close to the *EC*_50_ value of 1.2 μM (blue bars) and Δ*G*_E_ for the closed-flipped and flipped-open isomerisations (ochre bars), according to model F1. The blue lines indicate negative cooperativity for the binding steps and the ochre lines positive cooperativity for both isomerisations. (**b**) Same as a but for H319K ATP and with model F2. Compared to wt, Δ*G*_E_ for the closed-flipped isomerization in H319K is notably decreased at all degrees of liganding, thereby preserving the degree of cooperativity (purple bars and lines). (**c**) Comparison of experimental fATP binding to wt and H319K channels (data points, n = 3–6), obtained from a previous study^27^, superimposed by the binding computed by the ^4^global fit (continuous lines).

Knowledge of the rate constants, and thus all equilibrium constants, in model F1 allowed us also to estimate the Gibbs free energies for the association in the binding steps, Δ*G*_A_, as well as for the flip steps and closed-open isomerisations, Δ*G*_E_, according to

2 $$\Delta G_{A} = \, - RT\ln \, (L \times k_{x} /k_{x + 1} )$$

and

3 $$\Delta G_{E} = \, - RT\ln \, \left( {k_{x} /k_{x + 1} } \right),$$

respectively. *R* is the molar gas constant, *T* the absolute temperature and *L* the ligand concentration. Setting *L* to 1 μM, a value close to *EC*_50_ = 1.2 μM, the Δ*G*_A_ values were attributed to the individual binding steps of model F1 (blue bars) and the Δ*G*_E_ values to the flip or closed-open isomerisations (ochre bars) (Fig. 6a). There are four major results: (1) The absolute values of Δ*G*_A_ for related steps in the closed, flipped and open states markedly decrease, indicating that flipping and opening successively promote ligand binding. (2) Δ*G*_A_ increases with the degree of liganding for all, closed, flipped and open states, indicating a pronounced negative cooperativity in the binding process. With respect to the second binding step, the degree of Δ*G*_A_ increase for the third binding step is similar for the closed, flipped and open states. Such a negative cooperativity is very surprising because in P2X2 channels concentration-activation relationships are steep and generate typically Hill coefficients around 2, suggesting positive cooperativity. This cooperativity can therefore only be generated by subsequent reactions. (3) Accordingly, Δ*G*_E_ for the flip reaction strongly decreases with the degree of liganding, necessarily to a higher degree as compared to Δ*G*_A_. Hence, the steeper drop of Δ*G*_E_ for the flip steps at increased liganding, as compared to the corresponding rise of Δ*G*_A_, apparently overcompensates for the negative cooperativity in binding, resulting in an overall positive cooperativity for channel activation. (4) The positive cooperativity in the closed-open isomerisations is much less pronounced than in the flip reactions. Together, these results indicate that it is the flip reaction which introduces the pronounced positive cooperativity in the activation of P2X2 channels, thereby overbalancing the pronounced negative cooperativity in the binding steps.

When considering the respective process for H319K and ATP in terms of our model, the effect of the mutation is solely to lower the energy barriers for the flip reaction, thereby keeping the slope for Δ*G*_E_ (Fig. 6b).

### Relating the computed to experimental ligand binding

To further confirm our approach, the concentration dependence of ligand binding has been attributed to the ligand binding determined experimentally by recording the fluorescence intensity of the binding of fATP to P2X2 channels. The experimental concentration-binding relationships were obtained from a previous report for wt and H319K channels^27^ and the theoretical curves obtained from the ^4^global fit (Fig. 4a–e, Supplementary Table 6) were superimposed (Fig. 6c). Despite the fact that the experimental and the predicted relationships were obtained from significantly different conditions, fluorescence-optical analyses in cell cultures versus current recordings with the patch-clamp technique in isolated cells, the relationships match reasonably well.

## Discussion

We performed a detailed kinetic analysis of P2X2 receptors that was based on equilibrium and non-equilibrium data at multiple ligand concentrations, extended by data from the mutant H319K, positioned in the middle of the transmission pathway, and data obtained with the fluorescent ligand of reduced apparent affinity^27^. We investigated the resulting four data sets with four intimately coupled kinetic schemes (model F1-F4). These results allowed us to identify a surprising, but also simple, cooperativity pattern for wild type P2X2 channels and to recognize that the typically steep concentration-*P*_o_ relationship for these channels is the result of a positively cooperative flip reaction, overbalancing a negative cooperativity in ATP binding. Moreover, we identified a marked hysteresis of the net probability fluxes in an activation-deactivation cycle for the third binding step and that the facilitated opening in the H319K mutant is caused by a significantly enhanced flipping with only one ligand bound.

The result of coexisting negative cooperativity in the binding and positive cooperativity in the flip step shows that in P2X2 channels the subunits essentially interact already at the initial stages of activation and not only in the concerted closed-open reaction of the pore as suggested earlier^11^. This initial interaction is present in both the binding process itself and the transmission of the signal along the β-14 sheet as part of the conformational flip. Moreover, our results show that in wild type P2X2 channels relevant activation requires all three subunits, confirming on the one hand previous reports^10,13–15^, but conflicting on the other with reports suggesting that two subunits suffice for full activation^10,11^. The existence of an intermediate flip step in the activation pathway has already been suggested by three other studies using markedly different experimental approaches, for P2X2 channels with either a kinetic analyses^34^ or by using covalently bound 8-thiocyano-ATP^35^, as well as for P2X7 *and* P2X2 channels with photo-affinity labeling using 2(3)-*O*-(4-benzoylbenzoyl)-ATP^36^. It remains to be shown to what extent the flip characterized herein corresponds to the flip reported in these studies. Our study did not yield the necessity to include five discernable key steps in the activation process^18^. Usage of a model with three binding steps for trimeric channels is certainly plausible for a trimeric channel. Therein, however, the combination of pronounced negative cooperativity for ligand binding, i.e. the binding of a ligand inhibits the binding of the next ligand, and even more pronounced positive cooperativity for the flip reaction, i.e. the binding of the next ligand facilitates the flip more than the binding of the previous ligand, is a new molecular mechanism of cooperative activation gating in P2X channels. An increased binding affinity for intermediate flipped states compared to closed states has been shown previously for nicotinic acetylcholine^29^ and glycine receptors^37^.

The major methodological impact of our approach is that extended global fits with complex and intimately coupled kinetic schemes can be effectively used to study multiple data and that this approach enabled us to identify as much as 26 rate constants in model F1, topping all our previous approaches in CNG and HCN channels, even when using orthogonal binding data in addition to current data^24–26^. For CNGA2 channels this led to the identification of a cooperativity sequence in the cGMP binding of ‘negative–positive-no’ for the second, third and fourth binding step^24^ whereas for HCN2 channels this corresponding sequence was ‘positive–negative-positive’ for channels pre-activated by hyperpolarizing voltage^26^ and ‘negative-no-positive’ at depolarized voltages^38^. Based on global fits of ligand-induced activation and deactivation solely, as performed herein, we already previously showed for CNGA2 channels intricate cooperativity^22^, as we did by analyzing voltage-induced activation and deactivation time courses of tetrameric HCN2 channels^23^. Global fits provided also essentially new insights into the function of other ion channels, e.g. for the gating charge movement in Shaker channels^21^ and for the effects of permeating K^+^ ions on the *IV*-relationships of viral Kv channels^20^, all measured in ensemble currents and under equilibrium conditions. Also single-channel currents were subjected to global fit approaches, as shown elegantly e.g. for glycine receptors^39^. Furthermore, our extended global fit approach is promising to gain more insights in other P2X receptor subtypes for instance the enigmatic process of pore dilatation in P2X7 receptors^40,41^ or in the function of individual subunits in heteromeric assemblies^42^.

To some extent our extended global fit analysis is related to the widely used double-mutant cycle analysis^43^, determining Gibbs free energy differences for two single and the corresponding double mutant to specify free energies of interaction^44^. An often used approach for analyzing ion channels in macroscopic recordings is to determine *EC*_50_ values and translate them to free energies of interaction^45^. Compared to these analyses, our approach includes more extensive functional information from the channel than simply using *EC*_50_ values (1) by fitting activation and deactivation time courses in their concentration-dependence and also the steepness of the *P*_o_ relationship, (2) by using the combination of a mutation and a second ligand with different apparent affinity, and (3) by fitting a system of four intimately coupled kinetic schemes with three ligand binding sites which is physically much more meaningful compared to a simple *EC*_50_ value. Global fits were also used previously to thermodynamically characterize the function and interaction of proteins^46^, thereby modulating the proteins by mutations^47–49^ or by molecules functionally interacting with the proteins^50–52^. Another strategy is to globally fit orthogonal observables, such as e.g. calorimetric, spectroscopic or additional biosensing experiments^53^.

In conclusion, we provide a plausible hypothesis for the steep concentration-*P*_*o*_ relationship resulting from a negatively cooperative ATP binding countered by occupancy-dependent intermediate flip transitions. These results shed light into the conformational changes proceeding in the activation of P2X receptors.

## Materials and methods

### Synthesis of fATP

The synthesis of the novel fluorescent ATP derivative 2-[DY-547P1]-AET-ATP (fATP) has been described previously^27^.

### Molecular biology and cell culture

Rat wt P2X2 (RRID: Addgene_137071) and mutant P2X2 H319K (RRID: Addgene_137072) in pcDNA5/FRT/TO and derived stable cell lines (RRID: AC line CVCL_YJ33 for P2X2 wt, RRID: AC line CVCL_YJ34 for P2X2H319K) has been described previously (Sattler et al.). HEK293 cell lines containing an inducible promoter (Flp-In-T-REx 293, Invitrogen) were cultured in MEM supplemented with 10% FCS, non-essential amino acids (Gibco) and antibiotics according to the instructions of the manufacturers. Cell lines were used up to a passage number of 20. For most macroscopic measurements, cells were seeded on glass coverslips and used 24–48 h after tetracycline induction. For single-channel and macroscopic current recordings subjected to noise analysis, cells were used 2–6 h after induction to keep expression reasonably low.

### Electrophysiology

Recording of macroscopic currents was performed with a standard patch-clamp technique in the whole-cell mode^54^. The patch pipettes were pulled from borosilicate glass (ID 1.0 mm, OD 2.0 mm; Hilgenberg, Malsfeld, Germany) using a micropipette puller (P-97, Sutter Instrument, Novato, USA). The pipettes were filled with intracellular solution containing (mM) 142 NaCl, 5 BAPTA, 5 EGTA and 10 HEPES, pH 7.4. The pipette resistance was 2.5–6.0 MΩ. The bath solution contained (mM) 142 NaCl, 10 EGTA, 10 HEPES and 10 Glucose, pH 7.4. For current recording the cells were lifted from the chamber bottom by the patch pipette and positioned in front of the outlet of the application system.

Solution switches were carried out with a theta-glass pipette (inner diameter ~ 100 μm, WPI), mounted on a piezo device, or with a three-barrel glass (inner diameter ~ 600 μm, Warner Instruments), controlled by a step motor (SF-77B, Warner Instruments). The speed of the laminar solution flow out of the barrels was 2–5 cm/s. The time of the switch around a cell at the patch pipette was determined to be below 10 ms by switching between different salt solutions. This time was sufficiently short with respect to the current time courses. Saturation of activation was determined with ATP at 100 µM for wt P2X2 and 1 µM for P2X2 H319K. The currents were recorded with an Axopatch 200B or HEKA EPC 10 amplifier in combination with the ISO3 or Patchmaster and software. The sampling rate was either 2 or 10 kHz and the recordings were on-line filtered at 1 and 2.9 kHz, respectively, using a 4-pole Bessel filter. Recordings for noise analysis were sampled at 50 kHz and filtered at 10 kHz. The currents were recorded at a constant holding potential of − 50 mV.

Single-channel recordings were performed in the cell-attached configuration at − 50 mV. The pipettes were pulled from quartz tubing (ID 0.7 mm, OD 1.0 mm; Science Products, Hofheim, Germany) using a micropipette puller (P-2000, Sutter Instruments, Novato; USA). Pipettes were filled with intracellular solution (mM 142 NaCl, 5 BAPTA, 5 EGTA and 10 HEPES, pH 7.4) and an ATP concentration near the *EC*_50_ value (1 µM wt, 30 nM H319K). The pipette resistance was 10–15 MΩ. The resting potential of the cells was zeroed by the potassium chloride bath solution (mM 142 KCl, 10 EGTA, 10 HEPES, 10 glucose, pH 7.4 with KOH). Data were sampled at 20 kHz and on-line filtered at 5 kHz using a 4-pole Bessel filter.

### Conventional data evaluation

Concentration-activation relationships were constructed from the maximum currents during a solution application. These current amplitudes were normalized with respect to the current at saturating ATP (wt 100 µM; H319K 1 µM in each individual cell). In Fig. 1a the resulting data points were fitted with

4 $$I/I_{{{\text{max}}}} = {1}/\left( {{1} + \left( {EC_{{{5}0}} /\left[ {\text{X}} \right]} \right)^{H} } \right)$$

with the Origin 8.5.1 software using a non-linear curve fitting routine with statistical weighting. *I* is the actual current amplitude and *I*_max_ the maximum current amplitude at saturating ATP. *EC*_50_ is the ligand concentration generating half maximum current. *H* is the respective Hill coefficient. [X] is the actual ligand concentration of ATP.

The amplitude of the single-channel current was calculated from data filtered digitally down to 1 kHz and respective amplitude histograms were fitted with the sum of *j* = 2 Gaussian functions (Fig. 1b).

5 $$y = \mathop \sum \limits_{j = 1}^{2} A_{j} \exp \left( { - \frac{{\left( {i - i_{j} } \right)^{2} }}{{2\sigma_{j}^{2} }}} \right)$$

*A*_*j*_ is a calibration factor, *i* the amplitude of the actual single-channel current and *i*_*j*_the mean amplitude of the single-channel current for the closed or the open channel whose difference provides the mean single-channel current *i*_*s*_.

Experimental data are given as mean ± s.e.m.

### Global fit strategies

Each applied concentration pulse protocol consisted of a step to a defined concentration and, after a specified time, of a step back to zero concentration to evoke activation and deactivation time courses, respectively. The activation time courses from switching on the ligand concentration until the maximum and the deactivation time courses were analyzed. All time-dependent currents at the different concentrations were normalized to the respective current maximum to achieve in the fit for each current the same weight, even if the amplitude differed by orders of magnitude. These time courses were fitted together with steady-state activation relationships. Systems of first-order differential equations were used. Each differential equation describes the time-dependent change of the occupational probability *dp*_x_/*dt* of a considered state x resulting from the sum of all inputs and outputs.

This results in a system of *n* differential equations *dp*_x_/*dt* (x = 1…*n*). x denotes the n states of the model. Consider for example model F1 (Fig. 2b) with x running from C_0_ to O_3_:

6.a $$dp_{{{\text{C}}0}} /dt = k_{{2}} \times p_{{{\text{C1}}}} - L \times { 3}k_{{1}} \times p_{{{\text{C}}0}}$$

6.b $$dp_{{{\text{C1}}}} /dt = L \times { 3}k_{{1}} \times p_{{{\text{C}}0}} + {2}k_{{4}} \times p_{{{\text{C2}}}} + k_{{8}} \times p_{{{\text{F1}}}} - \, \left( {k_{{2}} + L\times {\text{ 2}}k_{{3}} + k_{{7}} } \right) \, \times p_{{{\text{C1}}}}$$

$$\begin{array}{*{20}c} {} & . & {} \\ {} & . & {} \\ {} & . & {} \\ \end{array}$$

6.j $$dp_{{{\text{O3}}}} /dt = L \times k_{{{25}}} \times p_{{{\text{O2}}}} + k_{{{21}}} \times p_{{{\text{F3}}}} {-} \, \left( {{3}k_{{{26}}} + k_{{{22}}} } \right) \, \times p_{{{\text{O3}}}}$$

*L* is the ligand concentration and *k*_a_ denotes a rate constant.

The system of differential Eqs. (6) can be written in a compact form in matrix notation as follows:

7 $$\frac{dp}{{dt}} = coef\left( L \right) \cdot p\left( t \right)$$

*p(t)* is the column vector of the probabilities to be in one of the states C_0_ to O_3_.

*coef(L)* is the matrix of the coefficients of the differential equation system depending on the concentration *L*. $\frac{dp}{dt}$ is the column vector of the derivatives of the probabilities.

An own program was developed with the Matlab software to fit the data. Because we didn’t use any constraints concerning the rate constants, the number of fit parameters was equal to the number of rate constants.

The system of differential Eqs. (6, 7) was solved numerically using the Eigenvalue method. As a result, we got for each fit point of the experimental curves numerical values of the occupation of states. The sum of the occupation of all open states is the open probability. In this way, the *P*_o_ vs. time courses and the steady-state *P*_o_ vs. concentration curves were calculated from the set of model parameters. Varying the model parameters step by step using a modified Levenberg–Marquardt algorithm^53^ the program finds an optimal set of parameters for a minimum difference between the experimental and the calculated curves.

The criterion for the best fit was the minimized sum of the squared deviations *S* according to

8 $$S={\sum }_{i=1}^{{n}_{t}}u{\sum }_{j=1}^{{n}_{d}}\frac{{\left({I}_{m}\left({t}_{ij}\right)-{I}_{c}\left({t}_{ij}\right)\right)}^{2}}{{\sigma }_{I}^{2}\left({t}_{ij}\right)}+{\sum }_{k=1}^{{n}_{s}}{s}_{k}{\sum }_{v=1}^{{n}_{x}}\frac{{\left({P}_{om}\left({x}_{kv}\right)-{P}_{oc}\left({x}_{kv}\right)\right)}^{2}}{{\sigma }_{Po}^{2}\left({x}_{kv}\right)}$$

The fitting process was terminated when the relative change of *S* in Eq. (8) from one iteration step to the next was below a set border.

*I*_m_(*t*_*ij*_) and *I*_c_(*t*_*ij*_) are the normalized measured and calculated current amplitudes of a fit point inside a trace *i* at a time *j*. *n*_t_ denotes the number of time traces, *n*_d_ the number of fit points in a trace, *n*_s_ the number of steady-state activation relationships used, and *n*_x_ the number of concentrations in a particular steady-state activation relationship. ${P}_{om}({x}_{kv})$ and ${P}_{oc}({x}_{kv})$ are the steady-state values of the measured and calculated open probabilities at a concentration *v *in a steady-state activation relationship *k*. ${\sigma }_{I}^{2}({t}_{ij})$ is the measured variance at time *j* of a fit point in a particular trace *i* (*i* = 1 … *n*_*t*_) and ${\sigma }_{\text{Po}}^{2}({x}_{kv})$ is the measured variance at concentration *v* in a particular steady-state activation relationship *k*. The squared deviations of the currents and the steady-state values in Eq. (8) were weighted by the reciprocal variances ${\sigma }_{I}^{2}({t}_{ij})$ and ${\sigma }_{\text{Po}}^{2}({x}_{kv})$ of the fit points to assign the better determined fit points a higher weight. The variances ${\sigma }_{I}^{2}({t}_{ij})$ and ${\sigma }_{\text{Po}}^{2}({x}_{kv})$ for each fit point were experimentally determined.

The weight of each curve in the fit process depends on the used number of fit points.

In the current time courses, the number of fit points was much bigger than that in the concentration-*P*_o_ relationships. Therefore, we employed in Eq. (8) the weighting factors *u and s*_*k*_ to give all time courses the same weight in the fit as all concentration-*P*_o_ relationships. By introducing these factors we achieved for all fits comparability of the errors of the fit parameters. The factors *u* and *s*_*k*_ were calculated such that the effective number of fit points of all current time courses equaled that of the concentration-*P*_o_ relationships. The effective number of fit points was obtained by the product of the number of fit points and the weighting factor. For a particular fit, *u* has the same value for all current time courses. *s*_*k*_ is a special factor for each particular concentration-*P*_o_ relationship correcting for the different number of fit points in the individual concentration-*P*_o_ relationships.

Hence, in all global fits we used 470 effective fit points for all time courses and 470 effective fit points for all concentration-*P*_o_ relationships.

As an example, the ^4^global fit employed n_t_ = 47 time traces and n_s_ = 4 steady-state relationships. We used for each time trace 100 fit points and for the steady-state relationships all experimentally determined data points (6, wt ATP; 4, wt fATP; 7, H319K ATP; 11, H319K fATP). Assigning each time trace the weighting factor u = 0.1 and the steady-state relationships the resulting weighting factors *s*_k_ = 19.59, 29.38, 16.79 and 10.68, respectively, we obtained the same number of 470 effective fit points for the time traces and for the steady-state relationships. This approach allowed us to directly compare the error of the resulting parameters of the fits even though they use different numbers of data points and data sets.

With the help of the *i*-th entry of the covariance matrix main diagonal (*cov*_*ii*_) the standard error for the *i*-th parameter *se(p*_*i*_*)* was calculated as

9 $$se\left({p}_{i}\right)= \sqrt{{cov}_{ii}}.$$

### Probability flux densities and transition pathway analysis

Using the constants obtained by the ^4^global fit with models 3, 3b, 3c and 3d (Supplementary Table 6), the time courses of the occupational probability *p*_X_(*t*) of the individual states X were computed for both activation and deactivation. From these time courses the unidirectional probability flux density from state X to an adjacent state Y was calculated by

10 $$f_{{\text{u,XY}}} \left( t \right)_{ } = p_{{\text{X}}} \left( t \right) \times k_{{{\text{XY}}}} ,$$

where *k*_XY_ is the rate constant specifying the transition from state X to state Y. The net probability flux density between these two adjacent states was obtained from the difference between the forward and backward unidirectional flux density according to

11 $$f_{{{\text{XY}} }} = f_{{\text{u,XY}}} - f_{{\text{u,YX}}} .$$

In model 3, a flux in the direction to O_3_ was assigned a positive sign. From the net probability flux densities, *f*_XY_, the total net probability fluxes, *F*_XY_, can be computed by the time integral over the time interval from the concentration jump (*t* = 0 s) to an end time, *t*_end_,

12 $$F_{{{\text{XY}}}} = \mathop \smallint \limits_{t = 0}^{{t_{{{\text{end}}}} }} f_{{{\text{XY}}}} dt.$$

For both, activation and deactivation, *t*_end_ was set to the end of the time courses used for fitting. As mentioned above we used for the fit process the activation curves from the beginning of the concentration jump until the maximum of the curve to ignore slow desensitization components. For the deactivation curves after removal of ATP, *t*_end_ was 5000 ms or 8000 ms. The resulting total net probability fluxes during activation and deactivation for the four data sets are shown in Fig. 5c–f.

## Supplementary Information

Supplementary Information.
