# Supplementary material for: Unravelling the intricate cooperativity of subunit gating in P2X2 ion channels
Source: Sci Rep. 2020 Dec 10;10:21751. doi: 10.1038/s41598-020-78672-w (PMC7729398; doi:10.1038/s41598-020-78672-w)
Supplement: Supplementary file 1 — Supplementary Information. [file 41598_2020_78672_MOESM1_ESM.pdf]

Supplementary Information to

Unravelling the intricate cooperativity of subunit gating in P2X2 ion channels

by

Christian Sattler, Thomas Eick, Sabine Hummert, Eckhard Schulz, Ralf Schmauder, Andrea Schweinitz, Christopher Unzeitig, Frank Schwede, Klaus Benndorf

Supplementary Figures

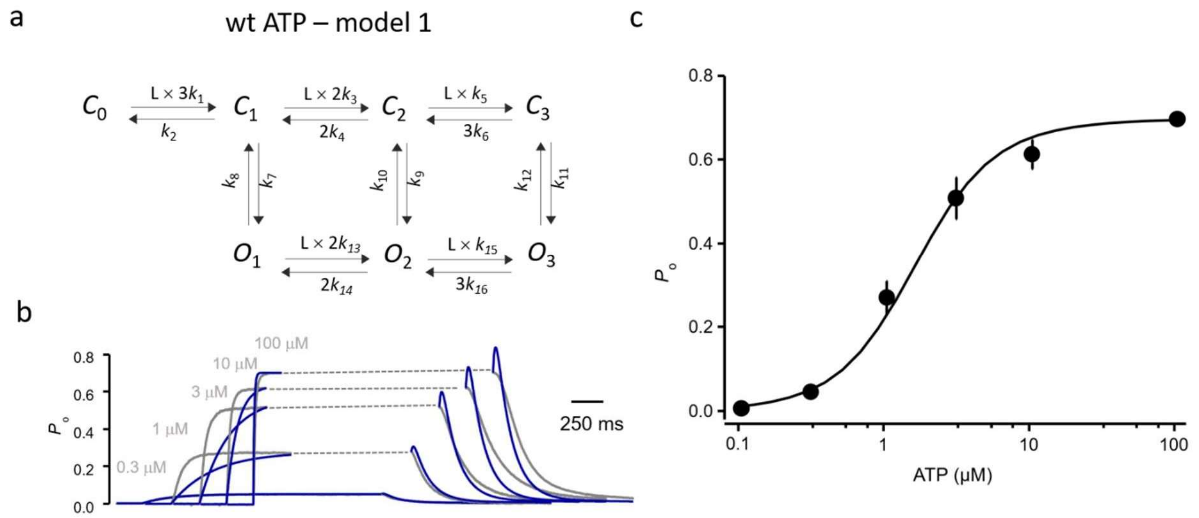

Supplementary Fig. 1 [1]Global fit to the data wt ATP with model 1. a Structure of the model. The model contains three ATP binding steps and direct closed-open transitions from the liganded states. b Time courses. Here, and also in Supplementary Figures 2-5, the gray traces represent the measured time courses and the blue curves were obtained by the respective fits. The model fails to describe the fast activation time courses at intermediate ATP concentrations and produces an upward deflection at early deactivation. c Equilibrium concentration-P_o relationships. The concentrations are provided by the legend to Fig. 4. For further explanation see text.

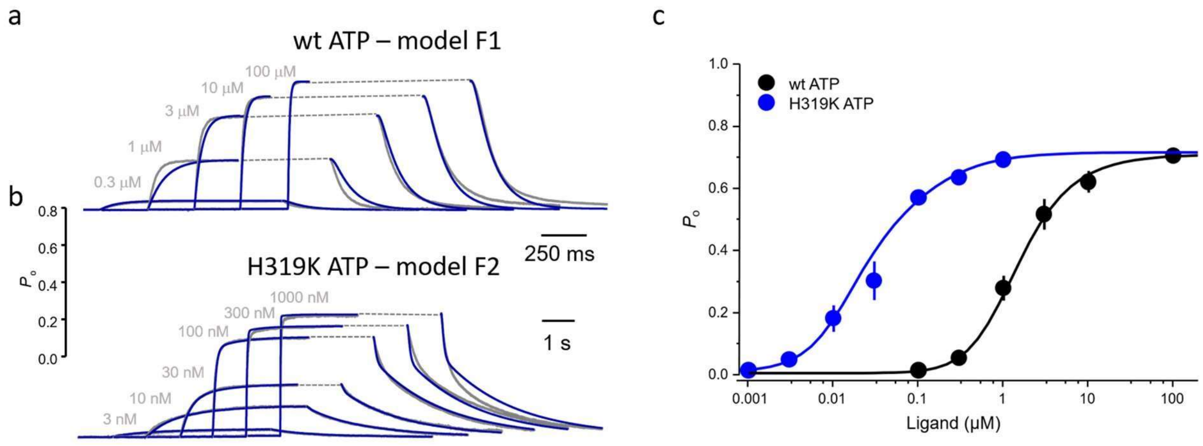

Supplementary Fig. 2 [2]Global fit to the data wt ATP and H319K ATP. a,b Time courses. C Equilibrium concentration-P_o relationships. The concentrations are provided by the legend to Fig. 4 and the values of the rate constants by Supplementary Table 2. For further explanation see text.

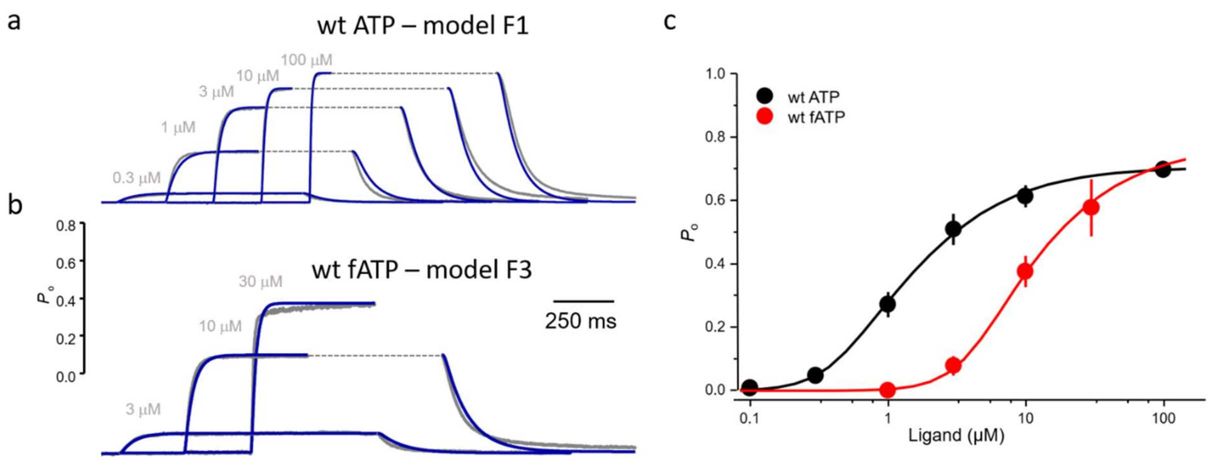

Supplementary Fig. 3 [2]Global fit to the data wt ATP and wt fATP. a,b Time courses. c Equilibrium concentration-P_o relationships. The concentrations are provided by the legend to Fig. 4 and the values of the rate constants by Supplementary Table 3. For further explanation see text.

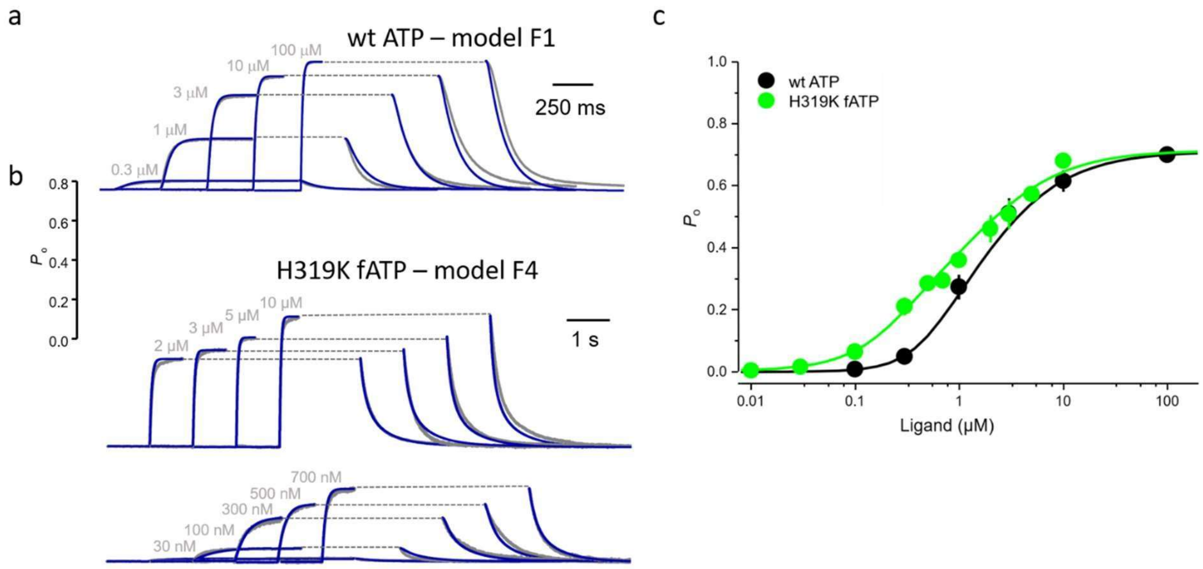

Supplementary Fig. 4 [2]Global fit to the data wt ATP and H319K fATP. a,b Time courses. c Equilibrium concentration-P_o relationships. The concentrations are provided by the legend to Fig. 4 and the values of the rate constants by Supplementary Table 4. For further explanation see text.

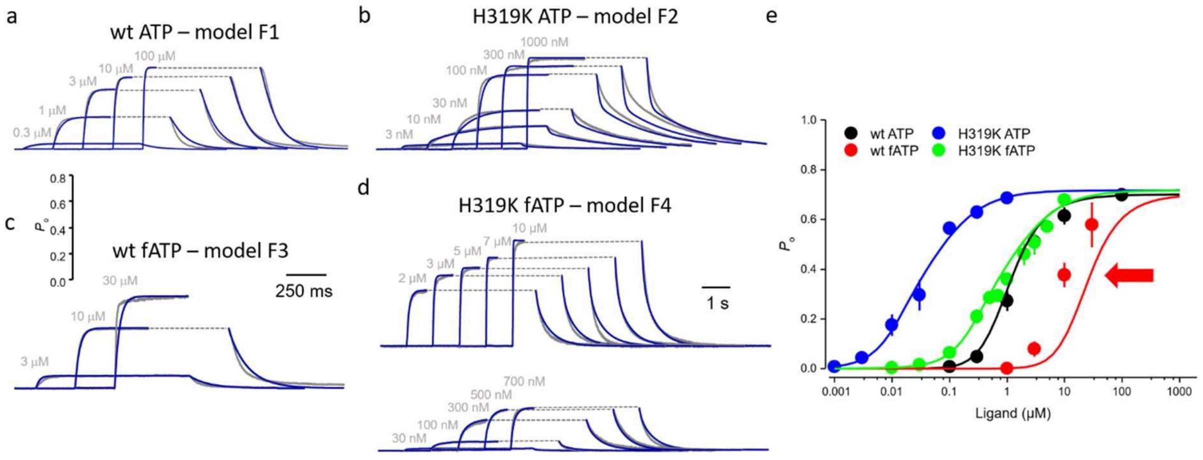

Supplementary Fig. 5 [4]Global fit to the data wt ATP, H319K ATP, wtf ATP, and H319K fATP. a-d Time courses. e Equilibrium concentration-P_o relationships. The concentrations are provided by the legend to Fig. 4 and the values of the rate constants by Supplementary Table 5. For further explanation see text.

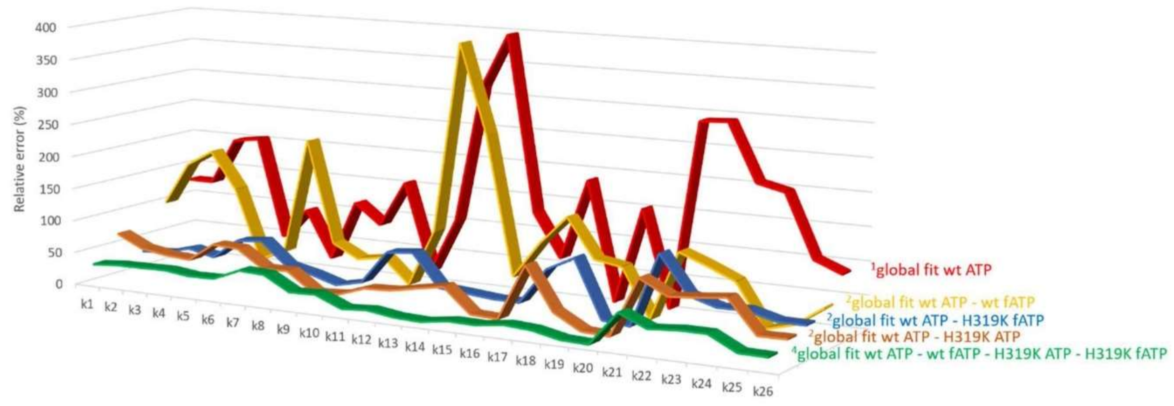

Supplementary Fig. 6 Improvement of relative error by extending the global fits. Compared are the errors of the individual rate constants for the [1]global fit (wt ATP data), three [2]global fits (either wt ATP and H319K ATP data, wt ATP and wt fATP data or wt ATP H319K fATP data), and the [4]global fit (wt ATP, H319K ATP, wt fATP, H319K fATP data). It should be noted that k_{21} and k_{22}, are much too fast with respect to our data and should therefore not be interpreted physically.

Supplementary Tables

[1]global fit		wt ATP	
Rate constant	Dimension	value	error %
k_1	$s^{-1}M^{-1}$	9.49E+07	126.43
k_2	s^{-1}	2.32E+02	126.03
k_3	$s^{-1}M^{-1}$	6.37E+08	197.46
k_4	s^{-1}	1.97E+02	202.50
k_5	$s^{-1}M^{-1}$	1.06E+07	49.70
k_6	s^{-1}	2.60E+01	96.17
k_7	s^{-1}	5.01E-02	23.37
k_8	s^{-1}	9.77E+00	115.87
k_9	s^{-1}	3.19E+01	85.21
k_{10}	s^{-1}	4.79E+02	155.48
k_{11}	s^{-1}	1.14E+02	20.98
k_{12}	s^{-1}	1.04E+02	105.04
k_{13}	$s^{-1}M^{-1}$	3.27E+10	314.77
k_{14}	s^{-1}	7.79E+02	397.08
k_{15}	$s^{-1}M^{-1}$	7.56E+07	129.49
k_{16}	s^{-1}	1.13E+01	63.59
k_{17}	s^{-1}	1.76E-01	187.09
k_{18}	s^{-1}	4.19E-01	6.28
k_{19}	s^{-1}	1.10E+01	152.11
k_{20}	s^{-1}	1.17E+01	5.42
k_{21}	s^{-1}	4.42E+12	289.39
k_{22}	s^{-1}	1.07E+12	291.77
k_{23}	$s^{-1}M^{-1}$	4.83E+04	206.16
k_{24}	s^{-1}	5.14E-04	196.53
k_{25}	$s^{-1}M^{-1}$	9.20E+08	102.62
k_{26}	s^{-1}	3.11E+01	85.78

Supplementary Table 1 Parameters of the [1]global fit wt ATP data. For further explanation see text.

^{2}global fit		wt ATP		H319K ATP	
**Rate constant**	**Dimension**	**value**	**error %**	**value**	**error %**
k_1	$s^{-1}M^{-1}$	2.03E+10	68.27	2.03E+10	68.27
k_2	s^{-1}	4.17E+03	49.60	4.17E+03	49.60
k_3	$s^{-1}M^{-1}$	2.03E+08	43.28	2.03E+08	43.28
k_4	s^{-1}	1.97E+02	40.39	1.97E+02	40.39
k_5	$s^{-1}M^{-1}$	1.83E+06	68.56	1.83E+06	68.56
k_6	s^{-1}	6.15E+01	66.59	6.15E+01	66.59
k_7	s^{-1}	5.52E-01	40.33	3.73E+00	77.18
k_8	s^{-1}	9.14E+02	41.05	1.37E+00	65.47
k_9	s^{-1}	6.03E+01	12.67	4.08E+02	49.52
k_{10}	s^{-1}	1.11E+03	14.82	1.66E+00	54.18
k_{11}	s^{-1}	2.32E+02	28.72	1.57E+03	65.57
k_{12}	s^{-1}	7.21E+00	30.52	1.08E-02	68.28
k_{13}	$s^{-1}M^{-1}$	1.05E+06	38.41	1.05E+06	38.41
k_{14}	s^{-1}	1.14E-02	45.78	1.14E-02	45.78
k_{15}	$s^{-1}M^{-1}$	2.04E+08	9.30	2.04E+08	9.30
k_{16}	s^{-1}	1.16E+01	7.30	1.16E+01	7.30
k_{17}	s^{-1}	1.27E+11	91.78	1.27E+11	91.78
k_{18}	s^{-1}	6.84E+11	27.70	6.84E+11	27.70
k_{19}	s^{-1}	7.57E+00	4.51	7.57E+00	4.51
k_{20}	s^{-1}	9.93E+00	2.54	9.93E+00	2.54
k_{21}	s^{-1}	7.93E+08	91.76	7.93E+08	91.76
k_{22}	s^{-1}	3.23E+08	69.91	3.23E+08	69.91
k_{23}	$s^{-1}M^{-1}$	6.68E+05	73.41	6.68E+05	73.41
k_{24}	s^{-1}	1.75E-03	78.14	1.75E-03	78.14
k_{25}	$s^{-1}M^{-1}$	8.66E+08	29.38	8.66E+08	29.38
k_{26}	s^{-1}	1.54E+01	29.75	1.54E+01	29.75
**Factors**		f =6.76 ± 2.49		g =1.50E-03 ± 5.91E-04	

Supplementary Table 2 Parameters of the ^{2}global fit to wt ATP and H319K ATP data. The green fields indicate where the factors f and g caused a difference in the rate constants of flipping. For further explanation see text.

^{2}global fit		wt ATP		wt fATP	
Rate constant	Dimension	value	error %	value	error %
k_1	$s^{-1}M^{-1}$	9.57E+08	98.46	2.05E+08	111.05
k_2	s^{-1}	2.33E+04	163.79	5.18E+04	174.56
k_3	$s^{-1}M^{-1}$	3.64E+10	187.52	7.81E+09	200.11
k_4	s^{-1}	1.09E+03	131.45	2.42E+03	142.22
k_5	$s^{-1}M^{-1}$	7.05E+06	24.18	1.51E+06	36.77
k_6	s^{-1}	1.23E+01	41.93	2.74E+01	52.69
k_7	s^{-1}	1.07E-03	219.14	1.73E-03	229.47
k_8	s^{-1}	4.49E+02	62.93	4.49E+02	62.93
k_9	s^{-1}	1.86E+01	42.06	3.02E+01	52.39
k_{10}	s^{-1}	5.60E+02	49.11	5.60E+02	49.11
k_{11}	s^{-1}	1.02E+02	11.82	1.66E+02	22.15
k_{12}	s^{-1}	3.80E+02	99.47	3.80E+02	99.47
k_{13}	$s^{-1}M^{-1}$	2.31E+09	388.29	4.95E+08	400.88
k_{14}	s^{-1}	4.94E-03	252.51	1.10E-02	263.27
k_{15}	$s^{-1}M^{-1}$	1.02E+08	42.94	2.19E+07	55.53
k_{16}	s^{-1}	2.20E+01	98.20	4.88E+01	108.97
k_{17}	s^{-1}	1.15E-03	141.31	1.15E-03	141.31
k_{18}	s^{-1}	8.10E-07	82.46	8.10E-07	82.46
k_{19}	s^{-1}	6.92E+00	75.69	6.92E+00	75.69
k_{20}	s^{-1}	1.11E+01	2.49	1.11E+01	2.49
k_{21}	s^{-1}	5.17E+12	107.23	5.17E+12	107.23
k_{22}	s^{-1}	4.53E+11	91.09	4.53E+11	91.09
k_{23}	$s^{-1}M^{-1}$	5.78E+06	72.81	1.24E+06	85.40
k_{24}	s^{-1}	2.82E-02	8.74	6.28E-02	19.50
k_{25}	$s^{-1}M^{-1}$	1.92E+09	20.06	4.11E+08	32.65
k_{26}	s^{-1}	2.25E+01	49.31	5.01E+01	60.08
**Factors**		$h = 0.21 \pm 0.03$		$j = 2.22 \pm 0.24$	
				$m = 1.61 \pm 0.17$	

Supplementary Table 3 Parameters of the ^{2}global fit to wt ATP and wt fATP data. The orange fields indicate where the factors h and j caused a difference in the rate constants of binding and unbinding, respectively. Dark blue indicates an additional factor m in the C_x->F_x transitions of model F3 fitting wt fATP data. For further explanation see text.

^{2}global fit		wt ATP		H319K fATP	
**Rate constant**	**Dimension**	**value**	**error %**	**value**	**error %**
k_1	$s^{-1}M^{-1}$	9.14E+07	29.39	6.66E+08	49.61
k_2	s^{-1}	3.80E+02	31.95	5.49E+03	52.00
k_3	$s^{-1}M^{-1}$	1.01E+08	42.76	7.34E+08	62.98
k_4	s^{-1}	3.58E+01	33.53	5.17E+02	53.58
k_5	$s^{-1}M^{-1}$	5.14E+05	63.97	3.75E+06	84.19
k_6	s^{-1}	4.06E+01	67.83	5.87E+02	87.88
k_7	s^{-1}	3.07E+01	33.46	1.16E+01	46.03
k_8	s^{-1}	2.02E+02	24.78	2.04E+00	50.15
k_9	s^{-1}	9.60E+01	12.74	3.64E+01	25.31
k_{10}	s^{-1}	1.54E+02	21.87	1.56E+00	46.46
k_{11}	s^{-1}	2.09E+02	72.37	7.92E+01	84.94
k_{12}	s^{-1}	3.06E+00	76.40	3.09E-02	101.77
k_{13}	$s^{-1}M^{-1}$	4.17E+06	27.52	3.04E+07	47.75
k_{14}	s^{-1}	3.61E-01	24.33	5.22E+00	44.37
k_{15}	$s^{-1}M^{-1}$	3.93E+07	20.60	2.86E+08	40.82
k_{16}	s^{-1}	2.84E+01	19.62	4.10E+02	39.67
k_{17}	s^{-1}	1.06E+04	74.49	1.06E+04	74.49
k_{18}	s^{-1}	4.52E+04	95.79	4.52E+04	95.79
k_{19}	s^{-1}	4.11E+00	8.66	4.11E+00	8.66
k_{20}	s^{-1}	1.16E+01	4.55	1.16E+01	4.55
k_{21}	s^{-1}	4.52E+11	116.15	4.52E+11	116.15
k_{22}	s^{-1}	1.82E+11	59.82	1.82E+11	59.82
k_{23}	$s^{-1}M^{-1}$	1.68E+04	47.00	1.22E+05	67.22
k_{24}	s^{-1}	9.65E-04	48.12	1.39E-02	68.17
k_{25}	$s^{-1}M^{-1}$	3.25E+08	37.07	2.37E+09	57.29
k_{26}	s^{-1}	3.34E+01	36.81	4.83E+02	56.86
**Factors**		f =0.38 $\pm$ 0.05		g =0.01 $\pm$ 2.48E-02	
		h =7.29 $\pm$ 1.47		j =1.45 $\pm$ 2.90	

Supplementary Table 4 Parameters of the ^{2}global fit to wt ATP and H319K fATP data. The green and orange fields indicate where the factors f and g as well as h and j caused a difference in the rate constants of flipping and binding/unbinding, respectively. For further explanation see text.

[4]global fit		wt ATP		H319K ATP		wt fATP		H319K fATP	
Rate constant	Dimension	value	error %	value	error %	value	error %	value	error %
k_1	$s^{-1}M^{-1}$	6.05E+08	16.74	1.71E+08	22.87	1.71E+08	22.87	6.05E+08	16.74
k_2	s^{-1}	1.92E+02	12.20	1.21E+03	18.24	1.21E+03	18.24	1.92E+02	12.20
k_3	$s^{-1}M^{-1}$	2.52E+08	8.89	7.14E+07	15.02	7.14E+07	15.02	2.52E+08	8.89
k_4	s^{-1}	2.72E+02	11.04	1.71E+03	17.08	1.71E+03	17.08	2.72E+02	11.04
k_5	$s^{-1}M^{-1}$	1.11E+07	15.12	3.14E+06	21.25	3.14E+06	21.25	1.11E+07	15.12
k_6	s^{-1}	8.81E+01	12.75	5.54E+02	18.79	5.54E+02	18.79	8.81E+01	12.75
k_7	s^{-1}	3.38E+02	33.40	7.67E+01	21.12	3.38E+02	33.40	3.38E+02	33.40
k_8	s^{-1}	7.89E+01	19.16	1.18E+05	9.75	7.89E+01	19.16	7.89E+01	19.16
k_9	s^{-1}	1.54E+02	21.34	3.50E+01	9.07	1.54E+02	21.34	1.54E+02	21.34
k_{10}	s^{-1}	2.68E-01	25.59	4.00E+02	10.19	2.68E-01	25.59	2.68E-01	25.59
k_{11}	s^{-1}	5.71E+02	18.37	1.29E+02	6.10	5.71E+02	18.37	5.71E+02	18.37
k_{12}	s^{-1}	7.32E-03	19.91	1.09E+01	10.51	7.32E-03	19.91	7.32E-03	19.91
k_{13}	$s^{-1}M^{-1}$	2.61E+07	6.46	7.39E+06	12.59	7.39E+06	12.59	2.61E+07	6.46
k_{14}	s^{-1}	2.09E-01	7.70	1.32E+00	13.74	1.32E+00	13.74	2.09E-01	7.70
k_{15}	$s^{-1}M^{-1}$	2.47E+08	8.02	6.98E+07	14.15	6.98E+07	14.15	2.47E+08	8.02
k_{16}	s^{-1}	1.45E+01	7.70	9.10E+01	13.74	9.10E+01	13.74	1.45E+01	7.70
k_{17}	s^{-1}	3.18E+00	11.17	3.18E+00	11.17	3.18E+00	11.17	3.18E+00	11.17
k_{18}	s^{-1}	1.65E+01	10.19	1.65E+01	10.19	1.65E+01	10.19	1.65E+01	10.19
k_{19}	s^{-1}	3.67E+00	4.61	3.67E+00	4.61	3.67E+00	4.61	3.67E+00	4.61
k_{20}	s^{-1}	8.29E+00	2.98	8.29E+00	2.98	8.29E+00	2.98	8.29E+00	2.98
k_{21}	s^{-1}	1.14E+12	20.81	1.14E+12	20.81	1.14E+12	20.81	1.14E+12	20.81
k_{22}	s^{-1}	4.48E+11	9.65	4.48E+11	9.65	4.48E+11	9.65	4.48E+11	9.65
k_{23}	$s^{-1}M^{-1}$	1.43E+05	14.36	4.06E+04	20.49	4.06E+04	20.49	1.43E+05	14.36
k_{24}	s^{-1}	5.00E-04	15.63	3.15E-03	21.67	3.15E-03	21.67	5.00E-04	15.63
k_{25}	$s^{-1}M^{-1}$	1.17E+09	13.35	3.31E+08	19.48	3.31E+08	19.48	1.17E+09	13.35
k_{26}	s^{-1}	1.19E+01	12.57	7.50E+01	18.61	7.50E+01	18.61	1.19E+01	12.57
**Factors**		f =4.41 ± 0.54		g =6.70E-04 ±1.03E-04		h =0.28 ± 0.02		j =6.30 ± 0.38	

Supplementary Table 5 Parameters of the [4]global fit to wt ATP, H319K ATP, wt fATP, and H319K fATP data. The green and orange fields indicate where the factors f and g as well as h and j caused a difference in the rate constants of flipping and binding/unbinding, respectively. For further explanation see text.

[4]global fit		wt ATP		H319K fATP		wt fATP		H319K fATP	
**Rate constant**	**Dimension**	**value**	**error %**	**value**	**error %**	**value**	**error %**	**value**	**error %**
k_1	$s^{-1}M^{-1}$	4.13E+08	30.67	4.13E+08	30.67	6.91E+07	35.02	6.91E+07	35.02
k_2	s^{-1}	1.33E+02	33.82	1.33E+02	33.82	7.42E+02	37.96	7.42E+02	37.96
k_3	$s^{-1}M^{-1}$	1.19E+08	34.43	1.19E+08	34.43	1.99E+07	38.78	1.99E+07	38.78
k_4	s^{-1}	1.03E+02	35.23	1.03E+02	35.23	5.74E+02	39.36	5.74E+02	39.36
k_5	$s^{-1}M^{-1}$	6.83E+06	30.54	6.83E+06	30.54	1.14E+06	34.89	1.14E+06	34.89
k_6	s^{-1}	8.94E+01	31.51	8.94E+01	31.51	4.98E+02	35.64	4.98E+02	35.64
k_7	s^{-1}	9.65E+01	48.58	1.08E+03	70.72	1.50E+03	54.33	1.08E+03	70.72
k_8	s^{-1}	2.96E+05	46.96	1.53E+02	84.30	2.96E+05	46.96	1.53E+02	84.30
k_9	s^{-1}	2.54E+01	25.01	2.84E+02	47.16	3.95E+02	30.77	2.84E+02	47.16
k_{10}	s^{-1}	5.01E+02	26.41	2.58E-01	48.16	5.01E+02	26.41	2.58E-01	48.16
k_{11}	s^{-1}	1.75E+02	10.01	1.96E+03	32.15	2.72E+03	15.76	1.96E+03	32.15
k_{12}	s^{-1}	1.38E+01	12.58	7.10E-03	49.92	1.38E+01	12.58	7.10E-03	49.92
k_{13}	$s^{-1}M^{-1}$	4.47E+07	6.91	4.47E+07	6.91	7.49E+06	11.26	7.49E+06	11.26
k_{14}	s^{-1}	2.50E-01	5.44	2.50E-01	5.44	1.39E+00	9.57	1.39E+00	9.57
k_{15}	$s^{-1}M^{-1}$	2.10E+08	11.52	2.10E+08	11.52	3.52E+07	15.87	3.52E+07	15.87
k_{16}	s^{-1}	1.10E+01	10.93	1.10E+01	10.93	6.12E+01	15.06	6.12E+01	15.06
k_{17}	s^{-1}	4.08E+00	16.44	4.08E+00	16.44	4.08E+00	16.44	4.08E+00	16.44
k_{18}	s^{-1}	2.57E+01	13.77	2.57E+01	13.77	2.57E+01	13.77	2.57E+01	13.77
k_{19}	s^{-1}	3.98E+00	5.15	3.98E+00	5.15	3.98E+00	5.15	3.98E+00	5.15
k_{20}	s^{-1}	9.00E+00	3.68	9.00E+00	3.68	9.00E+00	3.68	9.00E+00	3.68
k_{21}^{*}	s^{-1}	2.38E+11	54.20	2.38E+11	54.20	2.38E+11	54.20	2.38E+11	54.20
k_{21}^{**}	s^{-1}	2.38E+03	77.41	2.38E+03	77.41	2.38E+03	77.41	2.38E+03	77.41
k_{22}^{*}	s^{-1}	9.38E+10	36.21	9.38E+10	36.21	9.38E+10	36.21	9.38E+10	36.21
k_{22}^{**}	s^{-1}	9.38E+02	77.69	9.38E+02	77.69	9.38E+02	77.69	9.38E+02	77.69
k_{23}	$s^{-1}M^{-1}$	2.81E+05	39.74	2.81E+05	39.74	4.71E+04	44.09	4.71E+04	44.09
k_{24}	s^{-1}	5.63E-04	39.69	5.63E-04	39.69	3.13E-03	43.82	3.13E-03	43.82
k_{25}	$s^{-1}M^{-1}$	9.73E+08	18.21	9.73E+08	18.21	1.63E+08	22.56	1.63E+08	22.56
k_{26}	s^{-1}	8.86E+00	17.61	8.86E+00	17.61	4.93E+01	21.74	4.93E+01	21.74
**Factors**		f =11.20 $\pm$ 2.48		g =5.14E-04 $\pm$ 1.12E-04		h =0.17 $\pm$ 0.01		j =5.57 $\pm$ 0.23	
								m =15.59 $\pm$ 0.90	

Supplementary Table 6 Parameters of the [4]global fit to the data wt ATP, H319K ATP, wt fATP, and H319K fATP. The fit differs from that of Supplementary Fig. 6 by an additional factor m in the C_x->F_x transitions of model F3 fitting wt fATP data (dark blue fields). *Results for k_{21} and k_{22} from the initial [4]global fit. **Results for k_{21} and k_{22} after reduction of the rates under constant equilibrium until the simulated χ^2 for the [4]global fit increased by 1%.
